# Middle East and Central Asian dust reaches the South China Sea in summer

**DOI:** 10.1093/nsr/nwaf274

**Published:** 2025-07-08

**Authors:** Yu-Xiao Li, Li Luo, Jia-Wei Li, Shih-Chieh Hsu, Yuan-Zhe Ni, Shuh-Ji Kao

**Affiliations:** School of Ecology, Hainan University, China; State Key Laboratory of Marine Resources Utilization in South China Sea, Hainan University, China; State Key Laboratory of Marine Resources Utilization in South China Sea, Hainan University, China; College of Marine Science and Engineering, Hainan University, China; State Key Laboratory of Earth System Numerical Modeling and Application, Institute of Atmospheric Physics, Chinese Academy of Sciences, China; Research Center for Environmental Changes, Academia Sinica, China; Key Laboratory of Marine Environmental and Ecology, Ministry of Education, Ocean University of China, China; State Key Laboratory of Marine Resources Utilization in South China Sea, Hainan University, China

## Abstract

Mineral dust originating from the Middle East and Central Asia reaches the South China Sea in summer, potentially impacting N2-fixation.

The Middle East and Central Asian mineral dust contributes ∼30% of the global dust load [1], and is one of the primary contributors to global dust aerosols (including mineral dust, smoke, industrial pollutants etc.) [2]. Dust events in these regions peak in summer and model simulations suggest that most summer mineral dust is deposited in the Arabian Sea, Bay of Bengal and the tropical Indian Ocean, but a fraction can be transported eastward to the western Pacific Ocean, including the South China Sea (SCS) [1]. However, field observations confirming the arrival of Middle East and Central Asian dust in the SCS in summer are still lacking. As an oligotrophic sea, the SCS experiences N_2_ fixation limited by iron (Fe) or phosphorus (P) or Fe–P co-limitation [3–5]. Spring East Asian dust has been shown to enhance primary productivity in the SCS [5,6]. Summer biomass burning in Southeast Asia has been identified as a direct impact on marine aerosols in the SCS [7], leaving the origin, transport and deposition fluxes of dust aerosols, along with associated Fe and P, remaining poorly understood.

A 9-day super dust plume in June 2013 was captured by satellite datasets and by a cruise across the SCS, providing an opportunity to investigate the atmospheric dust sources, transport pathways and deposition fluxes in the summer SCS. Total suspended particulate samples (TSPs) were collected by the cruise from 17 to 29 June 2013 [Fig. 1a (blue line) and Fig. S1], the concentrations of water-soluble inorganic ions were measured by ion chromatography and trace elements were analyzed by inductively coupled plasma mass spectrometry (ICP-MS). To clarify the sources and transport pathways of summer dust in the SCS, we performed the models of air mass backward trajectories, chemical mass balance (CMB) and positive matrix factorization (PMF) and reanalyzed the dust optical depth (DOD) results from the Copernicus Atmosphere Monitoring Service (CAMS); the biomass burning activities in Southeast Asia were recorded by moderate resolution imaging spectroradiometer (MODIS) images, and the Visible Infrared Imaging Radiometer Suite (VIIRS). Dust plumes covering the Arabian Sea, Bay of Bengal, tropical Indian Ocean and SCS were also captured by the Cloud–Aerosol Lidar and Infrared Pathfinder Satellite Observations (CALIPSO) scanning images. Based on the observed datasets, the dry deposition fluxes of dust, Fe and P were calculated. In addition, the dust dry and wet deposition fluxes were also directly obtained from the Modern-Era Retrospective analysis for Research and Applications, Version 2 (MERRA-2) during our observed periods. All the detailed information about the methods can be found in Supplementary data (Text S1–S6).

**Figure 1.**
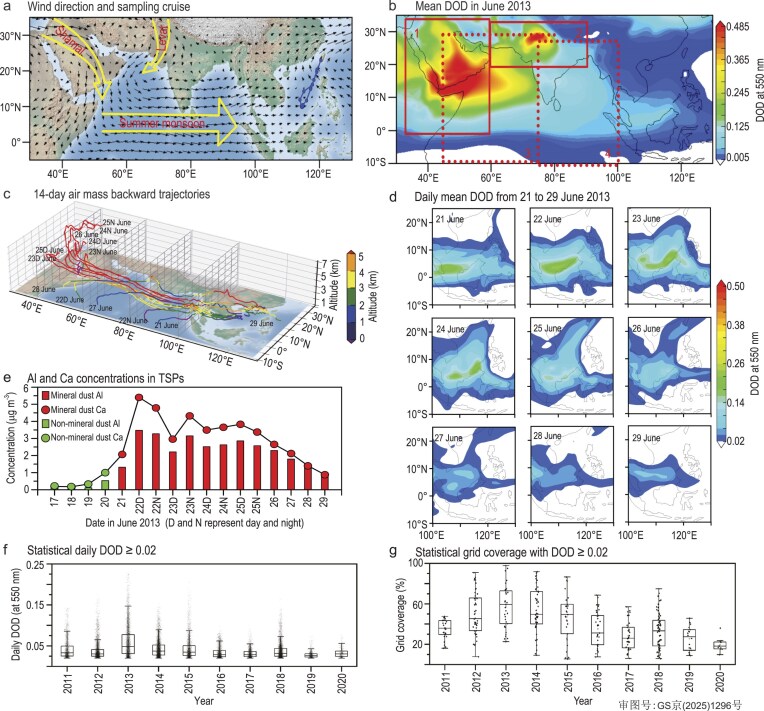
(a) Schematic of major wind systems (yellow hollow arrows: Shamal, Levar and Indian Summer Monsoon [8,9]) and 600 hPa wind field (black arrows) in June 2013, and the sampling cruise tracks (blue dotted line; details in Fig. S1 and Table S1). (b) Mean DOD in June 2013. Red solid boxes mark dust source regions (1: Middle East; 2: Central Asia [1,10]), while red dashed boxes denote dust outflow regions (3: Arabian Sea and western Indian Ocean; 4: Bay of Bengal and eastern Indian Ocean [12]). (c) 3D 14-day air mass backward trajectories. (d) Daily DOD over the SCS (from 21 to 29 June 2013). (e) Al and Ca concentrations in TSPs. (f) Daily DOD (dots) and (g) grid coverage (DOD ≥ 0.02) during summer dust days (2011–20) in the SCS. Large boxes show the interquartile range (25th–75th percentile), internal lines represent medians, and whiskers extend to the 10th and 90th percentiles.

In summer, the Shamal and Levar winds (Fig. 1a) lift mineral dust from the dust source regions [Arabian Peninsula (Fig. 1b, box 1) and Central Asia (Fig. 1b, box 2)] into the atmosphere, then these dust particles are transported southeastward to the dust outflow regions [Arabian Sea, Bay of Bengal and tropical Indian Ocean (Fig. 1b, boxes 3 and 4)] [8,9]. The CALIPSO scanning images also evidenced that the dust particles are transported in the atmosphere from the dust source regions southward to the tropical Indian Ocean (Fig. S2). Meanwhile, the Indian summer monsoon carries the dust particles across the Indian Ocean in a west-to-east direction [8,9]. The gradually decreased DOD from the west Indian Ocean to the SCS (Fig. 1b) and the CALIPSO scanning images (Figs S2 and S3) revealed the dust particles’ spatial distribution over the Indian Ocean and the widespread presence of dust aerosol in June 2013. Fourteen-day air mass backward trajectories showed that most of these air masses either originated at 5 km altitude over the Horn of Africa and Arabian Peninsula, or sourced from the Indian Ocean (Fig. 1c). The spatiotemporal overlap among all air mass backward trajectories (Fig. 1c), high DOD (DOD ≥ 0.02; Fig. 1d and Fig. S4) and the dust signature captured by the CALIPSO (Figs S2 and S3) indicated the influences of dust plumes over the Indian Ocean on TSPs sampled in the SCS. According to above discussion, we inferred that the mineral dust from the Arabian Peninsula (Fig. 1b, box 1, here defined as the Middle East by following [1]) and Central Asia (Fig. 1b, box 2, defined by [10]) can be transported southward to tropical Indian Ocean, and then eastward to the SCS in summer. Simulations from six global dust emission models reported that the mineral dust from the Horn of Africa, Middle East and Central Asia can reach the SCS in summer [1].

Concentrations of aluminum (Al) and calcium (Ca) (Al and Ca are widely considered to be useful tracers for indicating the impact of mineral dust on aerosols [11]) sharply increased from 20 June (0.53 μg m^−^^3^ for Al and 0.99 μg m^−^^3^ for Ca) to 21 June (1.32 μg  m^−^^3^ for Al and 2.06 μg m^−^^3^ for Ca), and remained at high levels until the end of the cruise on 29 June (Fig. 1e), indicating the influences of mineral dust on TSPs sampled from 21 to 29 June in the summer SCS. Therefore, we classified the TSPs collected from 21 to 29 June as dust TSPs, and the others (sampled from 17 to 20 June) as non-dust TSPs.

The CMB and PMF models were employed to quantify the relative contributions of the mineral dust to TSPs during the summer SCS cruise. The results showed that the mineral dust contributes the highest percentages (35.8%–40.4%, Figs S5 and S6) to dust TSPs, emphasizing the important contributions of mineral dust to the summer SCS dust TSPs. The reanalysis of DOD from 2011 to 2020 (Fig. 1f and g, Fig. S7) implied the frequent occurrence of summer dust aerosols in the SCS. The dust days (here defined as the grid coverage of DOD(≥0.02) >5% of the total 587 grids in the SCS) ranged from 11 to 73 in the summer SCS from 2011 to 2020 (Fig. S8), further illustrating the persistent influence of mineral dust on SCS TSPs.

Although an intense smog over Sumatra was observed on 19 June 2013 (Fig. S9) and high-density fire points were recorded from 18 to 25 June 2013 by satellite over Indonesia (Fig. S10), the results of CMB and PMF models showed the contributions of biomass burning aerosol to dust TSPs were 1.6% and 13.8%, which were obviously lower than the contributions (35.8%–40.4%) of mineral dust to dust TSPs during this cruise (Figs S5 and S6). In addition, the ratios of K/Al vs K/Ca (Fig. S11) in dust TSPs were much closer to crust rather than the biomass burning aerosols, suggesting less biomass burning influence compared to mineral dust input for dust TSPs during this observation.

The dust dry deposition fluxes in the SCS, calculated using fixed deposition velocities (7.1–27.4 kg km⁻^2^ d⁻^1^, average 15.7 ± 6.7 kg km⁻^2^ d⁻^1^), were significantly higher than those from the MERRA-2 reanalysis data (0.14–0.87 kg km⁻^2^ d⁻^1^, average 0.43 ± 0.23 kg km⁻^2^ d⁻^1^, Fig. S12). A similar discrepancy was observed in the Northeastern Arabian Sea, where observation-based fluxes (60–132 kg km⁻² d⁻¹, average 91.0 ± 23.5 kg km⁻² d⁻¹) [13] greatly exceeded MERRA-2 reanalysis data (1.5–18.4 kg km⁻^2^ d⁻^1^, average 5.0 ± 3.7 kg km⁻^2^ d⁻^1^). These findings align with a synthesis study reporting that local observation-based dust dry deposition fluxes are an order of magnitude higher than model simulations [14]. As important nutrients for N_2_ fixation in the SCS, the Fe and P deposition fluxes of dust TSPs (16.6 ± 5.4 and 2.3 ± 0.8 μmol m^−^^2^ d^−^^1^, from 21 to 29 June 2013) were 10.0 and 3.5 times higher than those during the non-dust TSPs (1.3 ± 0.9 and 0.7 ± 0.2 μmol m^−2^ d^−1^, from 17 to 20 June 2013, Table S3), indicating the important contribution of mineral dust to Fe in summer SCS.

Based on the stoichiometric ratios of N:Fe in N_2_-fixation phytoplankton [15] and the calculated seawater-soluble Fe fluxes in the summer SCS during dust days from 21 to 29 June, the estimated N_2_-fixation fluxes (52 ± 17 μmol m^−2^ d^−1^) were comparable to *in situ* measurements (45 ± 47 μmol m^−2^ d^−1^) during summer (Fig. S13). These findings suggest the dust-derived Fe supports N_2_ fixation in the summer SCS. Taking dust wet deposition into account (Fig. S4), the total dust deposition may bring more Fe to induce N_2_-fixation fluxes in the summer SCS.

In summary, by combining the reanalysis of satellite datasets and receptor models with chemical compositions in TSPs, we found that the Middle East and Central Asian dust can reach the SCS in summer. These dust plumes brought abundant Fe to the SCS, potentially stimulating the N_2_ fixation. This study offers an important understanding of intercontinental dust transport and reveals its potential impact on marine ecology thousands of kilometers away. Future work should pay more attention to the influences of Middle East and Central Asian dust on specific biogeochemical processes, such as N_2_ fixation, which was limited by Fe and/or P mostly, in the SCS.

## Supplementary Material

nwaf274_Supplemental_File
