# Supplementary material for: Middle East and Central Asian dust reaches the South China Sea in summer
Source: Natl Sci Rev. 2025 Jul 8;12(8):nwaf274. doi: 10.1093/nsr/nwaf274 (PMC12365750; doi:10.1093/nsr/nwaf274)
Supplement: nwaf274_Supplemental_File [file nwaf274_supplemental_file.pdf]

Supplementary materials for "Middle East and Central Asian dust reaches the South China Sea in summer"

Yu-Xiao Li[1,2], Li Luo[2,3]*, Jia-Wei Li[4], Shih-Chieh Hsu[5], Yuan-Zhe Ni[6], Shuh-Ji Kao[2]*

[1] School of Ecology, Hainan University, Haikou, 570228, China

[2] State Key Laboratory of Marine Resources Utilization in South China Sea, Hainan University, Haikou, 570228, China

[3] College of Marine Science and Engineering, Hainan University, Haikou, 570228, China

[4] State Key Laboratory of Earth System Numerical Modeling and Application, Institute of Atmospheric Physics, Chinese Academy of Sciences, Beijing, 100029, China

[5] Research Center for Environmental Changes, Academia Sinica, Taipei, China

[6] Key Laboratory of Marine Environmental and Ecology, Ministry of Education, Ocean University of China, Qingdao, 266100, China

*Corresponding authors: Professor Li Luo (Email: L.Luo@hainanu.edu.cn) and Professor Shuh-Ji Kao (Email: sjkao@hainanu.edu.cn)

Contents of this file

1. Text S1 to S6

2. Figure S1 to S14

3. Table S1 to S3

Text S1. Sampling and chemical analysis

Total suspended particulate samples (TSPs) were collected using a high-volume air sampler (Tisch Environmental, Inc.) with Whatman®41 cellulose filters (8″×10″) (Whatman Limited, Maidstone, UK) in this study. TSPs were collected atop the ship's bow to minimize exhaust interference. Sampling occurred only during cruising, with ship emissions contributing <3% to TSPs [1]. Detailed sampling information can be found in Table S1.

Water-soluble ionic concentrations (Na^+, Mg^{2+}, Ca^{2+}, K^+, NH_4^+, Cl^-, SO_4^{2-} and NO_3^-) were analyzed by ion chromatography (Dionex). A model ICS-1100 with conductivity detector (ASRS-ULTRA) and suppressor (ASRS-300) for anions (Cl^-, SO_4^{2-} and NO_3^-), and a model ICS-900 with conductivity detector (ASRS-ULTRA) and suppressor (CSRS-300) for cations (Na^+, Mg^{2+}, Ca^{2+}, K^+ and NH_4^+).

The concentrations of trace elements (P, Mg, K, Ca, Al, Fe, Ti, Y, Co, Mn, Cd, Ni, V, Cr, Zn, As, Pb) in TSPs were analyzed by ICP-MS (Inductively Coupled Plasma Mass Spectrometer, Elan 6100, Perkin-Elmer TM Instruments, United States). Detailed steps can be found in Hsu et al. [2–4]. Briefly, one-eighth of the filter was digested using 4 mL HNO_3 + 2 mL HF acid mixture in an ultra-high throughput microwave digestion system. Digestion efficiency was verified using a standard reference material (SRM1648, Urban Particulate Matter, National Institute of Standards and Technology (NIST), USA) subjected to the same treatment as the samples.

Text S2. Air mass backward trajectory

Air masses backward trajectories during the sampling periods were analyzed by using the Hybrid Single-Particle Lagrangian Integrated Trajectory (HYSPLIT) model developed by the National Oceanic and Atmospheric Administration (NOAA; http://www.arl.noaa.gov). The HYSPLIT model analysis is based on the Global Data Assimilation System (GDAS) data provided by the National Centers for Environmental Prediction (NCEP, https://ftp.arl.noaa.gov) with a spatial resolution of 1°×1°. In this study, the altitude for the backward trajectories were 1000 m and the backward times

were 14 days [1,5,6].

Text S3. Chemical mass balance (CMB)

To quantify the contributions of different emission sources to the TSPs, we run the CMB model (Version 8.2, the US Environmental Protection Agency, https://www.epa.gov/). The relationships between the mass concentrations of chemical species in TSPs and those chemical compositions emitted from the different emission sources can be presented by equation (S1).

$$C_i = \sum_{j=1}^{p} a_{ij} F_{ij} S_j \qquad (S1)$$

where C_i is the concentration of species *i* measured at the receptor samples (μg m^{-3}); α_{ij} is the coefficient of species *i* generated or removed during the transport process; F_{ij} is the mass fraction of species *i* in the chemical profile of the source *j* (%); S_j is the mass concentration of all species at the receptor samples assigned to the source *j* (μg m^{-3}). In this study, the CMB source profiles were selected based on previous studies conducted in the South China Sea [7–10], and eight potential emission sources were selected. The detailed parameters of CMB can be found in Table S2. Due to lack of TSPs and organic matter (OM) concentrations, we reconstructed these values using the previous empirical formula. The OC and EC concentrations were calculated by following Song et al [11], who reported a significant positive relationship between nss-K and OC/EC in the summer SCS during biomass burning periods in southeast Asia. The OM concentration is typically 1.4–2.2 times of OC concentration [12–15], thus, a conversion factor of 1.8 was used in this study. The concentration of mineral crust was calculated by dividing the concentration of Al by 0.084, representing the average Al content (8.04%) in Earth's crust. Finally, the TSPs concentrations were estimated as the sum of OM, mineral crust, sea salt, and secondary inorganic ions. Due to nss-K sources from both mineral dust and biomass burning, the OC and TSPs concentrations may have been overestimated.

Text S4. Positive Matrix Factorization (PMF)

The PMF 5.0 (US EPA 2013) is an effective source apportionment receptor model that does not require source profiles before analysis and has no limitation on source numbers [16–18]. In this study, EPA PMF Version 5.0 was used to determine the source apportionment of each element. In PMF 5.0, the number of samples and various compositions are regarded as an $n \times m$ of X (*ij*) matrix, and the observed mass concentration (X*ij*) of the *j*-th composition in the *i*-th sample can be expressed as:

$$x_{ij} = \sum_{k=1}^{p} g_{ik} f_{kj} + e_{ij} \qquad (S2)$$

$$X = GF + E \qquad (S3)$$

where *i* is the sample; *j* is the composition; *k* is the number of pollution sources; g_{ik} is the relative contribution of the *k*-th source to the *i*-th sample; f_{kj} is the composition content of *j* in the *k*-th emission source; and e_{ij} is the residual between the mass concentration and its analytical value of *j* composition in the *i*-th sample. Matrix X ($i \times j$) consists of the source contribution matrix G ($i \times k$), source component spectrum matrix F ($k \times j$) and residual matrix E ($i \times j$). The PMF model defines the sum of squares of the ratio of residual error to uncertainty (u_{ij}) of all samples as the objective function Q:

$$\mathrm{Q} = \sum_{i=1}^{n} \sum_{j=1}^{m} \left[\frac{x_{ij} - \sum_{k=1}^{p} g_{ik} f_{kj}}{u_{ij}} \right]^2 \qquad (S4)$$

The PMF model takes the non-negative values of elements in the pollution source contribution spectrum G and the pollution source composition spectrum F as the constraint conditions, seeks the solution of the minimization of objective function Q through the weighted least square method, and then determines G and F. Finally, after importing the sample data of different sampling periods in the model, the number of model operations was set to 20. The five potential emission sources identified in this study were consistent with those reported by previous aerosol observations in the SCS [19–23].

Text S5. Dust dry deposition fluxes

The dust dry deposition fluxes in the SCS were calculated by equation (S5),

$$F_{dust} = V_f \times C_f + V_c \times C_c \qquad (S5)$$

where V_f and V_c represent the fine (particle size < 2.5 μm) and coarse (particle size > 2.5 μm) dust deposition velocities, C_f and C_c is the dust concentration in fine and coarse particles, respectively. Due to V is a function of meteorological parameters (wind speed and RH), particle size and sea surface roughness [24]. According to previous model results, the dry deposition velocities of aerosol vary by more than 3 orders of magnitude for particles size ranging from 0.1 to 100 μm [25]. During our observation period, the wind speed ranged from 1.2 to 9.5 m s^{-1} (Table S1), and we only sampled the TSPs, it was difficult to provide variable dry deposition velocities. Thus, assumptions were made based on existing knowledge regarding fine and coarse particles dry deposition velocities. Based on both modeling and experimental results of aerosol deposition to the sea surface [24,25], dry deposition velocities of 0.1 cm s^{-1} for fine (particle size < 2.5 μm) and 1.0 cm s^{-1} for coarse (particle size > 2.5 μm) particles have been widely used in previous studies [26–32].

$$C_f = C^T \times f_f \qquad (S6)$$

$$C_c = C^T \times f_c \qquad (S7)$$

where f_f and f_c are the fractions of Al in fine and coarse dust, were given to 0.23 and 0.77 based on our observations in the SCS (Luo et al., unpublished data), respectively. C^T is the dust concentration and estimated by equation (S8),

$$C^T = \frac{C_{Al}}{8.04\%} \qquad (S8)$$

where C_{Al} is the concentrations of Al in TSPs. The 8.04% is the mass ratio of Al to the upper continental crust [33].

Note that, due to the uncertainties of fine and coarse dry deposition velocities, our calculated dust deposition fluxes in the summer SCS may have been over/under estimate.

Text S6. Fe and P dry deposition fluxes

The dry deposition fluxes of Fe and P in TSPs were calculated by equation (S9):

$$F_i = V_i^f \times f_i^f \times C_i^T + V_i^c \times f_i^c \times C_i^T \qquad (S9)$$

where V^f_i and V^c_i are the fine and coarse particle deposition velocities of element *i*, respectively. Based on the previous model and experimental results [24,25], aerosol deposition velocity to the sea surface was given to 0.1 cm s^{-1} for V^f_i (particle size < 2.5 μm) and 1.0 cm s^{-1} for V^c_i (particle size > 2.5 μm) as discussed above. The f^f_i and f^c_i are the fractions of element *i* in fine and coarse particles. According to our observations in the SCS, the f^f_{Fe} and f^c_{Fe} were 0.28 and 0.72, and the f^f_P and f^c_P were 0.52 and 0.48 (Luo et al., unpublished data), respectively. C^T_i is the total concentration of element Fe and P in TSPs.

The deposition fluxes of seawater-soluble Fe (F_{Fe}^{ss}) were calculated by equation (S10):

$$F_{Fe}^{ss} = F_{Fe}^{i} \times S_{Fe}^{i} \qquad (S10)$$

where F_{Fe}^{i} is the dry deposition flux of Fe in fine and coarse particles, S_{Fe}^{i} is the Fe solubility of fine and coarse particles in seawater [35].

Data Availability Statement

The dataset of air mass backward trajectory was based on the Global Data Assimilation System (GDAS) provided by the National Centers for Environmental Prediction (NCEP) (http://www.arl.noaa.Gov/index.php) and mapped using the Hybrid Single-Particle Lagrangian Integrated Trajectory (HYSPLIT) model developed by the National Oceanic and Atmospheric Administration (NOAA; http://www.arl.noaa.gov). Hourly wind field data was extracted from the ERA5 reanalysis (https://cds.climate. copernicus.eu/). The dust optical depth (DOD) was obtained from the Copernicus Atmosphere Monitoring Service (CAMS; http://atmosphere.copernicus.eu) and mapped using the Geographic Information System (GIS)-based software (MeteoInfo). The Visible Infrared Imaging Radiometer (Suite VIIRS) (https://firms.modaps.eosdis.

nasa.gov), MODIS images (http://earthobservatory.nasa.gov), CALIPSO satellite scanning images (https://www-calipso.larc.nasa.gov)， and the Modern-Era Retrospective analysis for Research and Applications, Version 2 (MERRA-2, https://gmao.gsfc.nasa.gov/reanalysis/) were also used in this study.

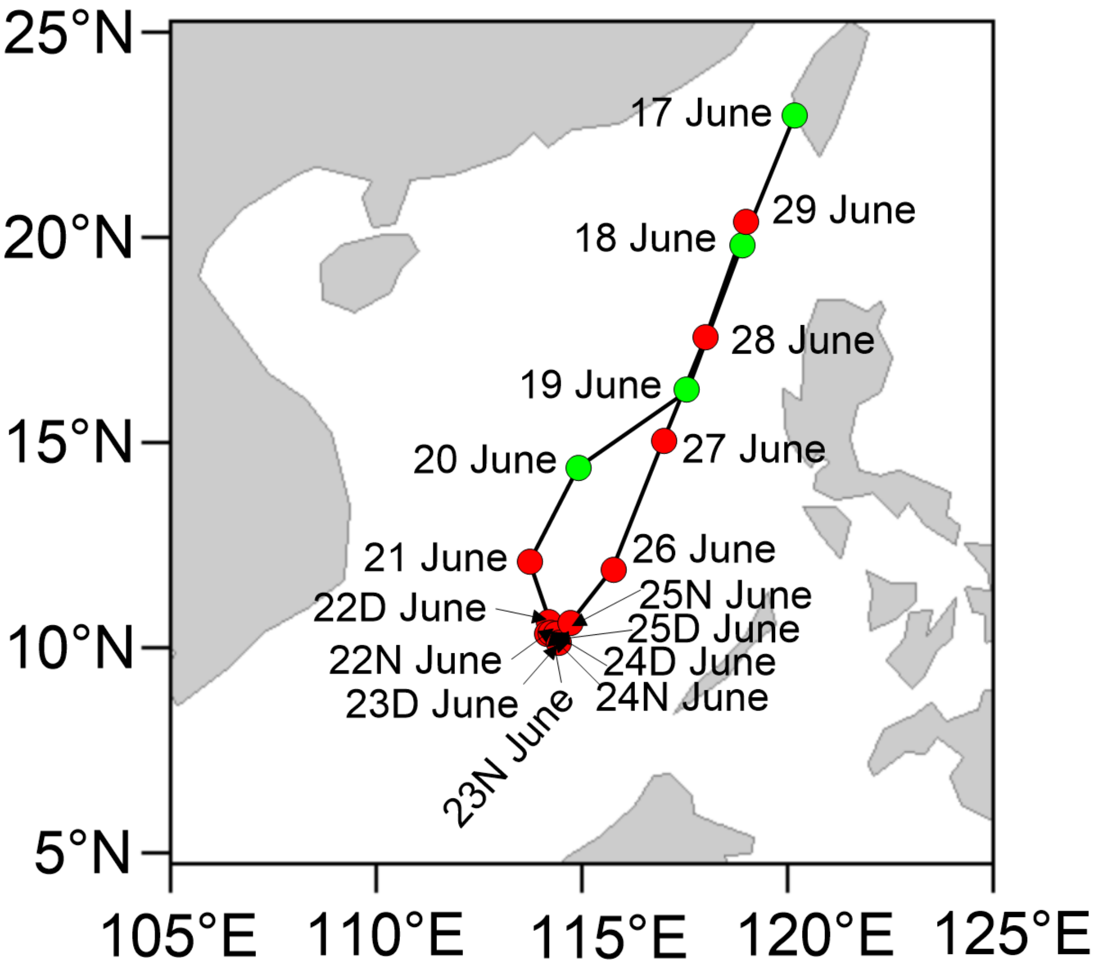

Figure S1 Sampling cruise (Detailed sampling information can be found in Table S1).

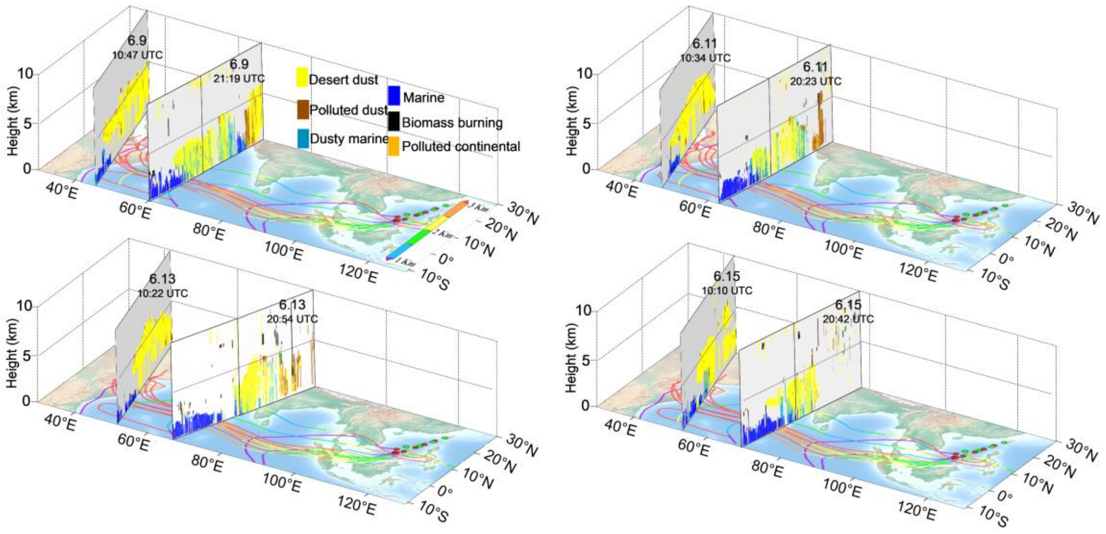

Figure S2 CALIPSO scanning images on 9,11,13 and 15 June, and 14 days air masses backward trajectories for dust TSPs.

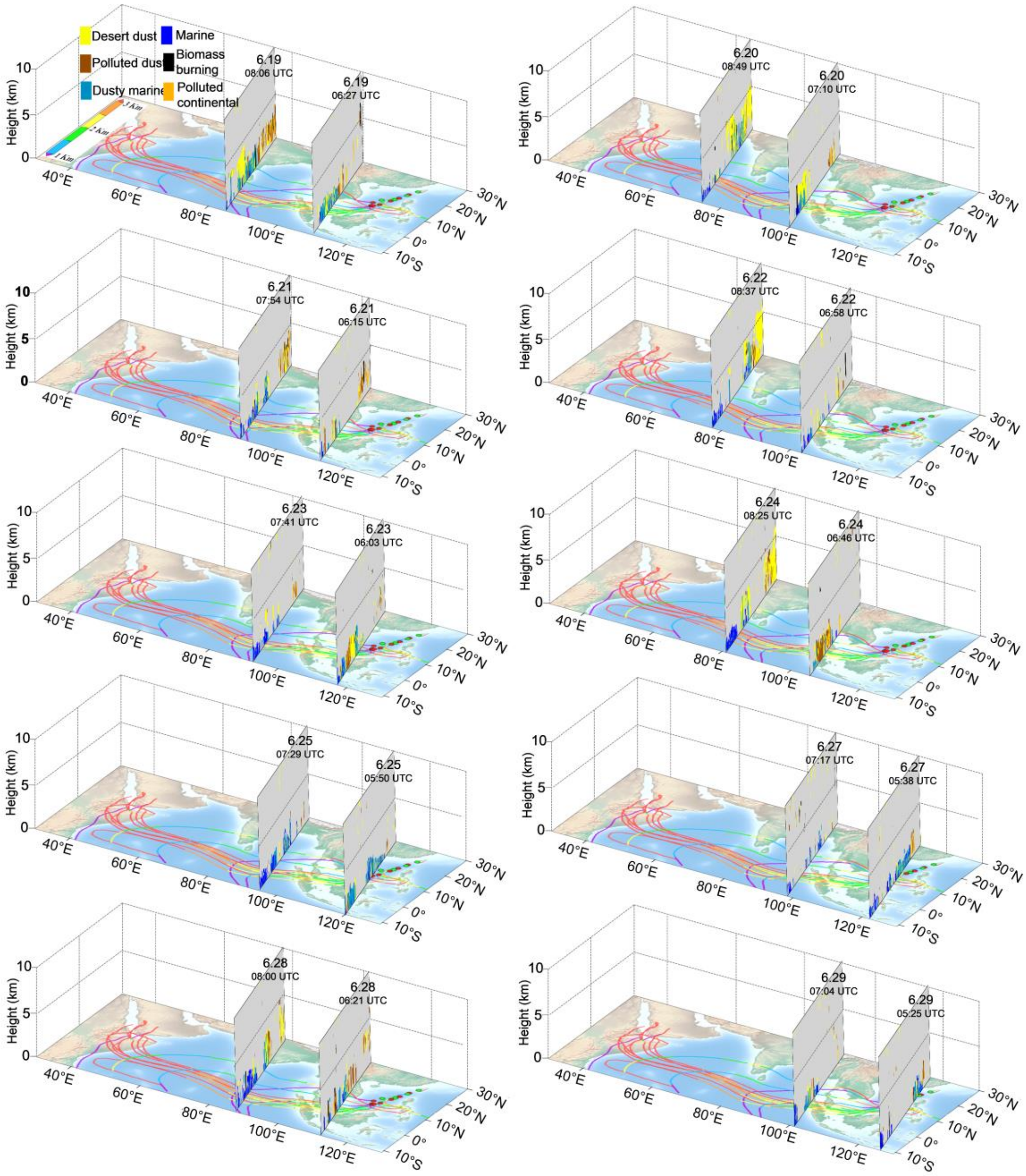

Figure S3 CALIPSO scanning images and 14 days air masses backward trajectories for dust TSPs.

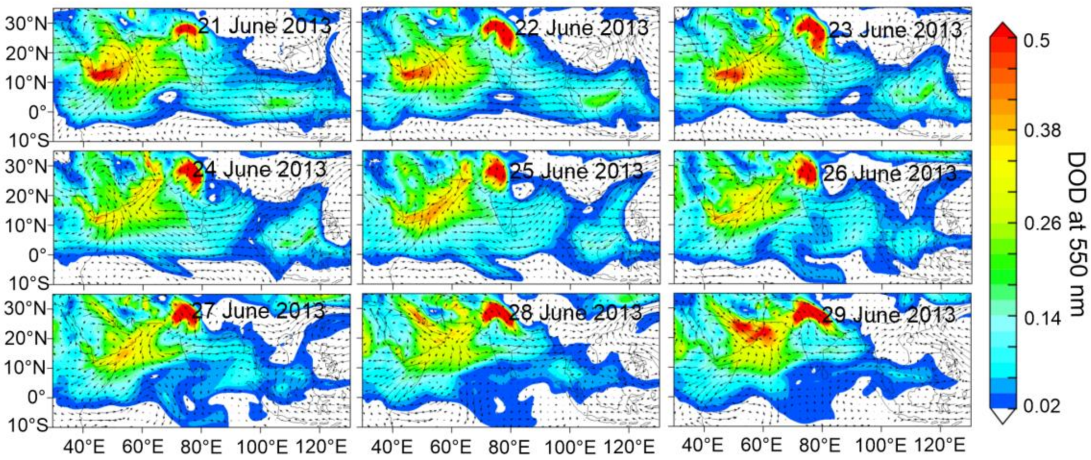

Figure S4 Daily mean DOD from 21 to 29 June 2013.

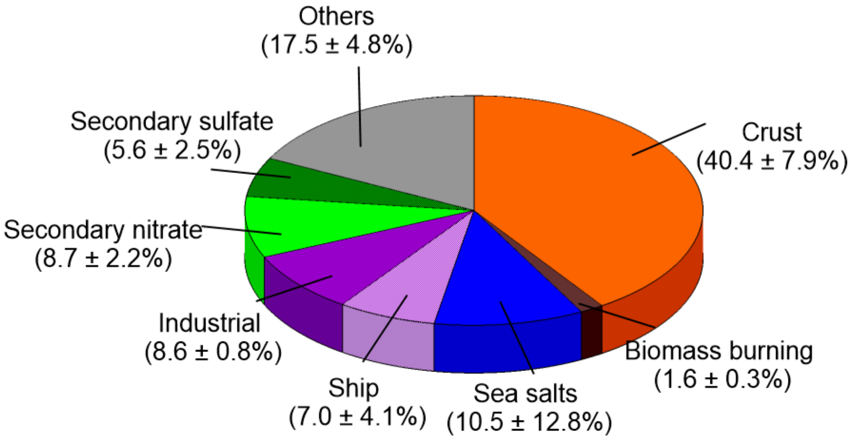

Figure S5 Contributions of different sources to dust TSPs by CMB model.

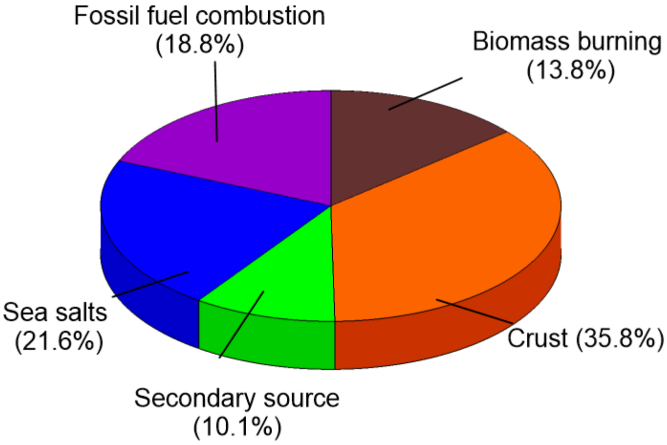

Figure S6 Contributions of different sources to dust TSPs by PMF model.

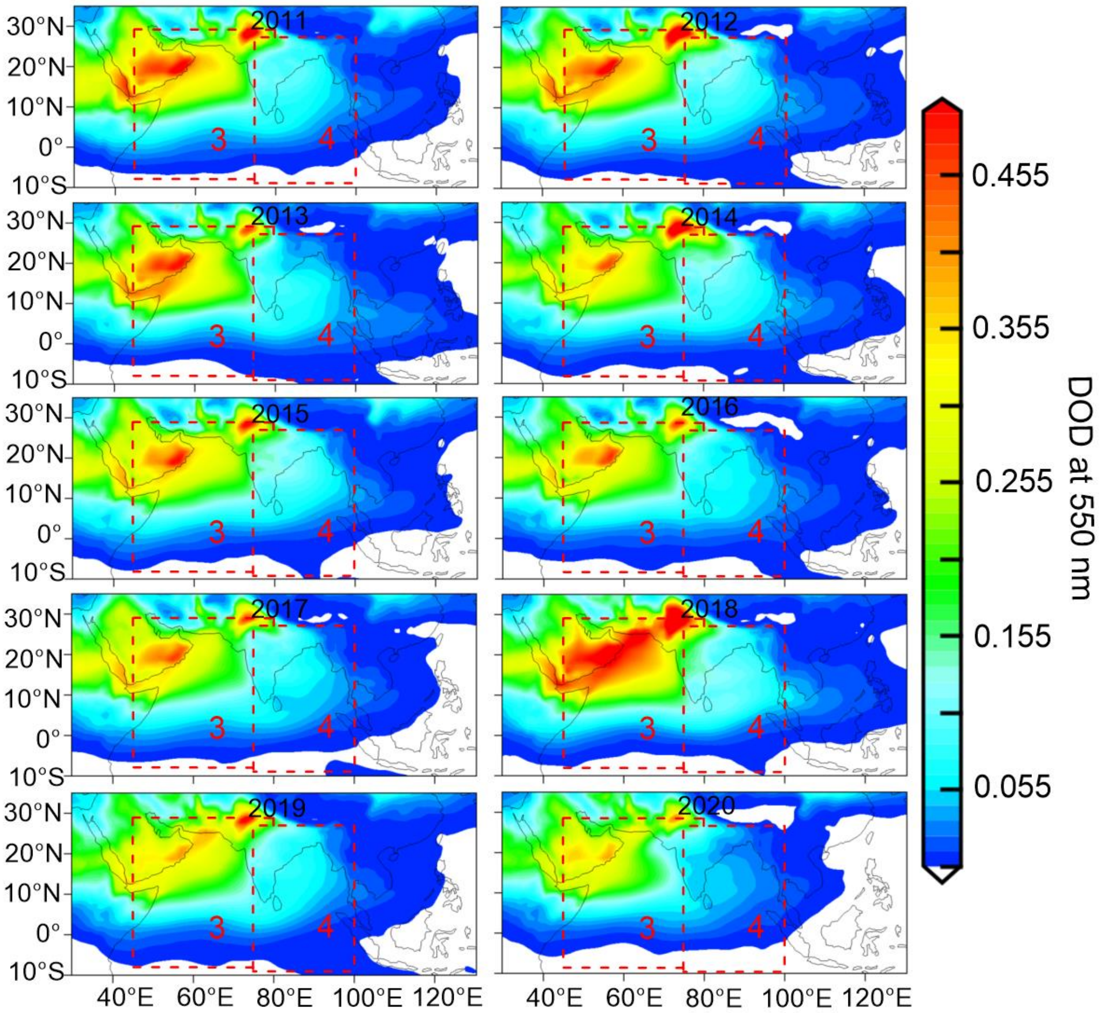

Figure S7 Summer (June, July and August) mean DOD from 2011 to 2020 (red dashed boxes denote dust outflow regions (3: Arabian Sea and western Indian Ocean; 4: Bay of Bengal and eastern Indian Ocean).

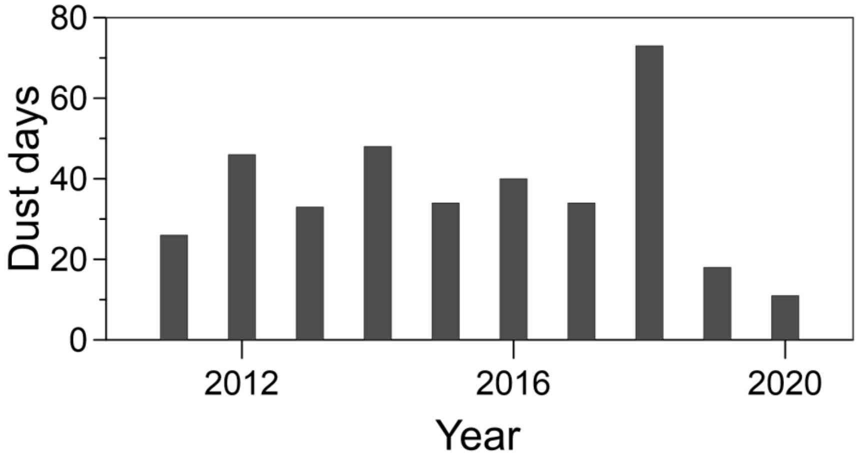

Figure S8 The statistical dust days in summer from 2011 to 2020.

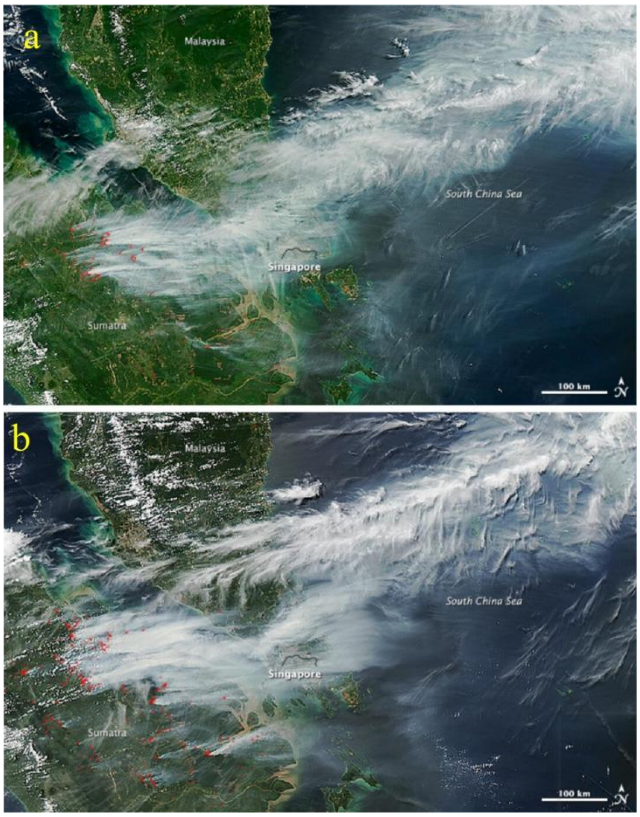

Figure S9 MODIS images in Sumatra and Indonesia on the morning (a) and afternoon (b) of 19 June 2013, showing the smog reached the south of SCS. http://earthobservatory.nasa.gov/IOTD/view.php?id=81431

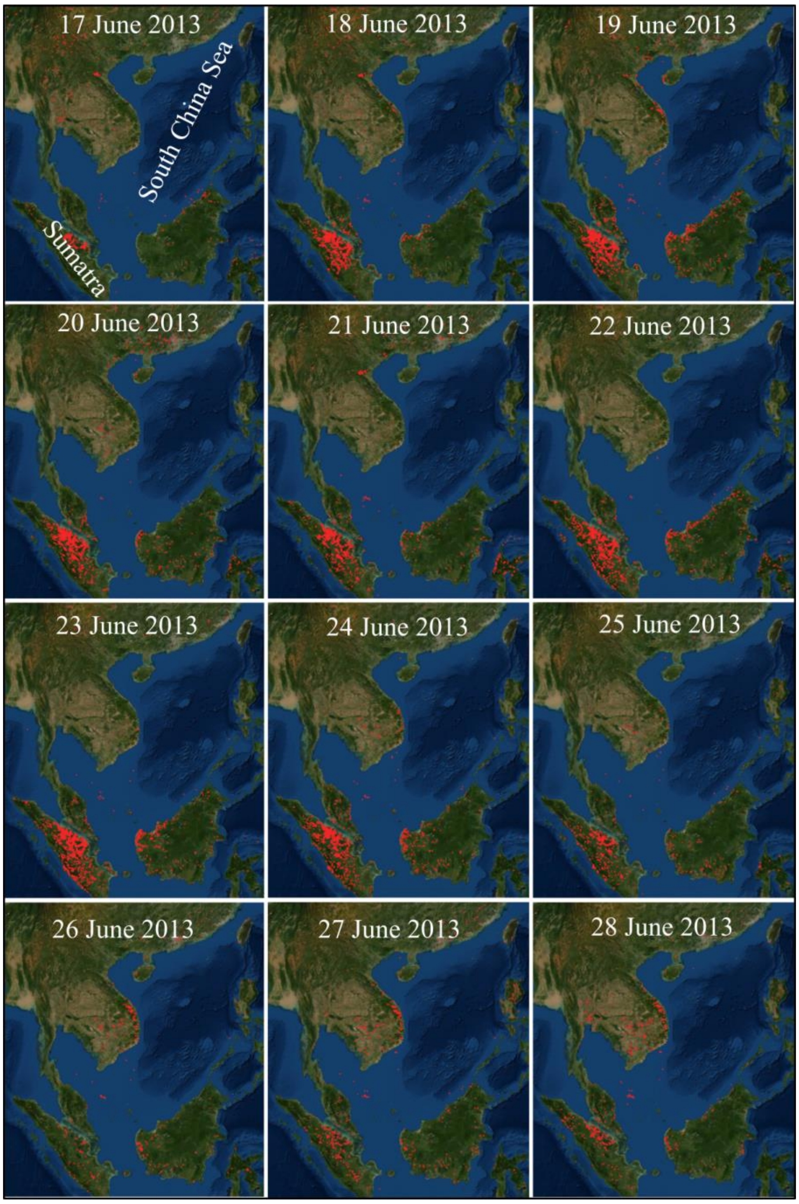

Figure S10 The fire points over the Southeast Asia from 17 to 28 June 2013.
https://firms.modaps.eosdis.nasa.gov/map/

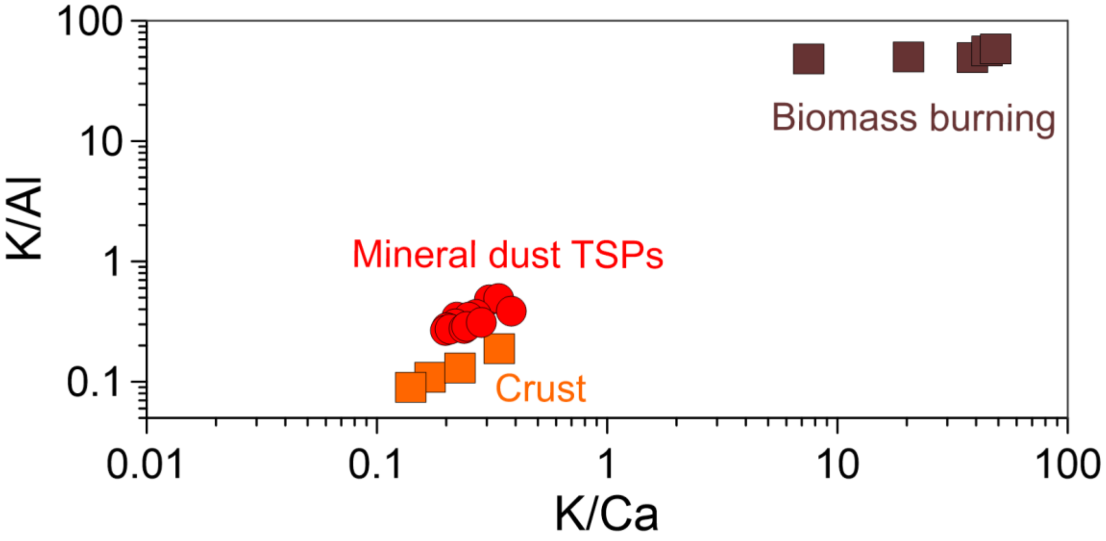

Figure S11 The ratios of K/Al and K/Ca in crust [33], dust TSPs and biomass burning aerosols [34].

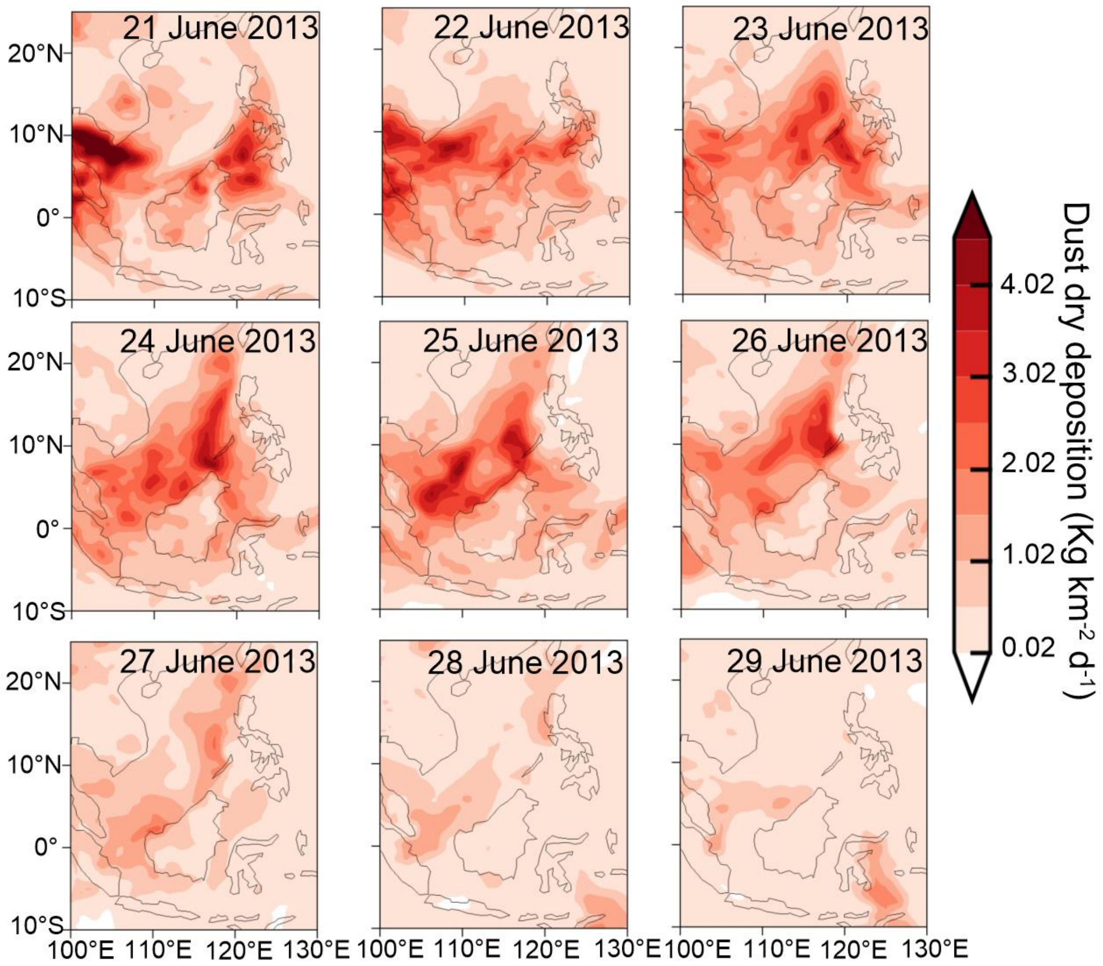

Figure S12 The dust dry deposition fluxes based on MERRA-2 reanalysis data.

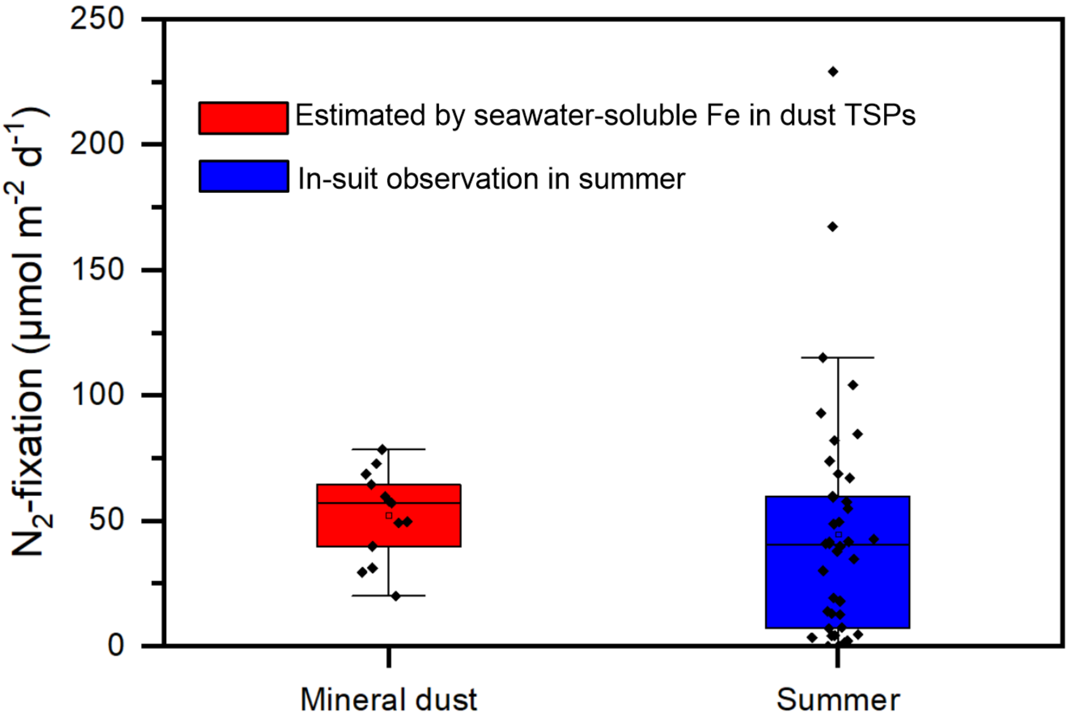

Figure S13 Comparison of N_2-fixation fluxes estimated by seawater-soluble Fe in dust TSPs (red box) with previous in-situ measurements (blue box) in the summer SCS [36–45].

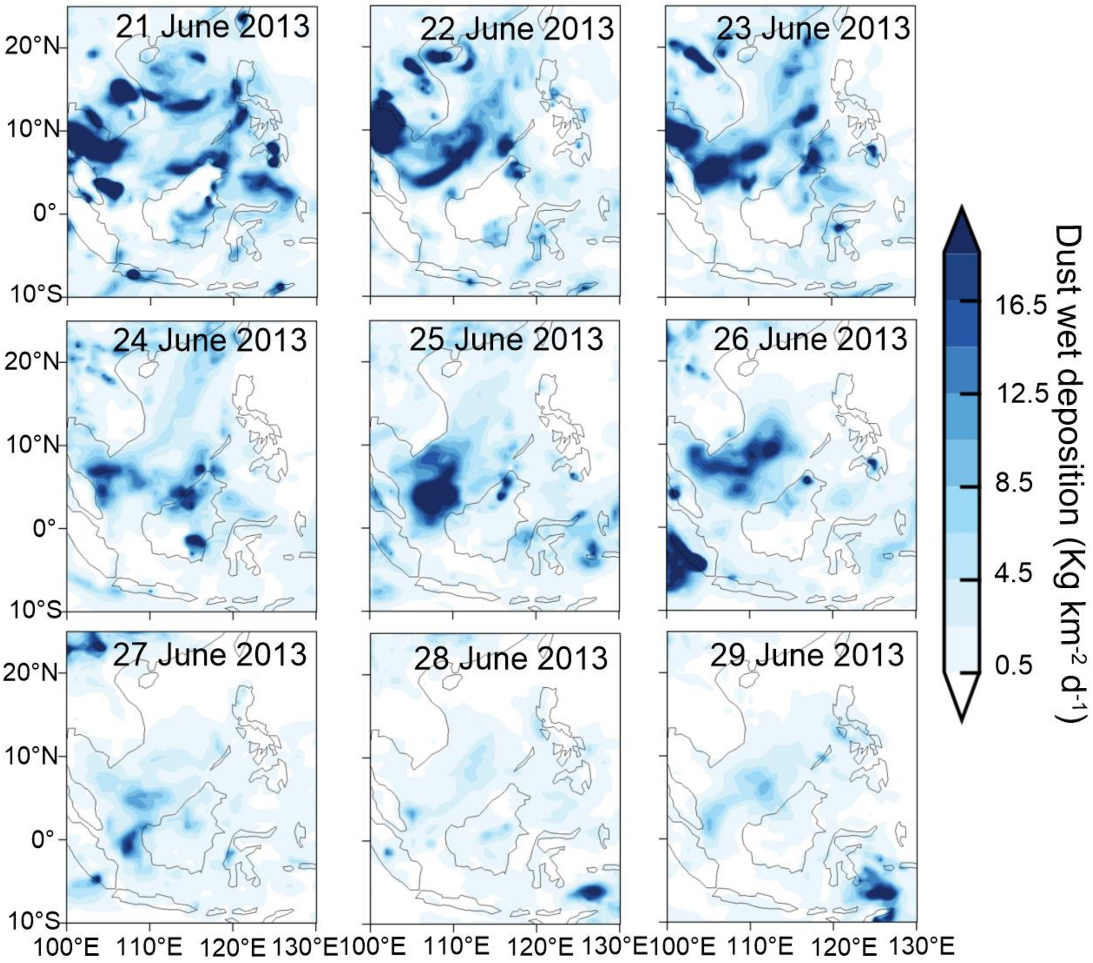

Figure S14 The dust wet deposition fluxes based on MERRA-2 reanalysis data.

Table S1 Sample information.

Types	Samples	Start time	End time	Longitude(°E)	Latitude(°N)	Wind speed	Wind direction
Non-dust	0617	2013/6/17 8:15	2013/6/18 7:59	120.10.25	22.58.23	1.20	171.03
	0618	2013/6/18 8:01	2013/6/19 8:15	118.54.00	19.49.10	2.47	163.27
	0619	2013/6/19 8:17	2013/6/20 8:03	117.32.41	16.18.39	4.54	53.25
	0620	2013/6/20 8:05	2013/6/21 8:03	114.53.46	14.26.05	7.86	348.38
Dust	0621	2013/6/21 8:05	2013/6/22 8:16	113.43.55	12.08.24	8.28	39.14
	0622D	2013/6/22 8:18	2013/6/22 20:01	114.14.48	10.36.00	9.54	59.89
	0622N	2013/6/22 20:04	2013/6/23 8:18	114.22.06	10.27.59	5.49	86.92
	0623D	2013/6/23 8:20	2013/6/23 20:03	114.10.37	10.22.59	5.90	80.71
	0623N	2013/6/23 20:05	2013/6/24 8:13	114.17.46	10.22.24	5.03	130.50
	0624D	2013/6/24 8:18	2013/6/24 20:00	114.19.11	10.23.23	4.92	126.39
	0624N	2013/6/24 20:02	2013/6/25 8:25	114.26.23	10.10.38	1.22	98.34
	0625D	2013/6/25 8:27	2013/6/25 20:10	114.19.50	10.24.57	3.21	136.82
	0625N	2013/6/25 20:12	2013/6/26 8:13	114.42.42	10.38.50	2.80	62.41
	0626	2013/6/26 8:16	2013/6/27 8:18	115.45.33	11.55.06	1.26	73.65
	0627	2013/6/27 8:22	2013/6/28 8:18	116.58.45	15.05.05	3.73	42.40
	0628	2013/6/28 8:20	2013/6/29 8:23	117.59.35	17.35.16	2.53	70.60
	0629	2013/6/29 8:28	2013/6/30 8:55	118.59.32	20.23.48	1.62	36.38

D and N represent day and night.

Table S2 Source profiles are applied for resolving the source apportionment of TSPs by using the CMB model.

Codes	Abbreviates	Emission Sources	References
SCT004	PBPRI1	Petroleum Cracking Plant	[46]
SCT007	PP004	Industrial Boilers (Oil)	[47]
SCT008	PP005	Industrial Boilers (Coal)	[47]
SCT009	PETRO1	Petroleum Industry	[46]
SCT010	STEEL1	Steel Industry	[48]
SCT011	STEEL2	Coke Plant	[48]
SCT012	STEEL3	Sinter Plant	[48]
SCT020	CEMENT	Cement Industry	[49]
SCT023	VEHICLE2	Vehicular Exhausts	[50]
SCT024	VEHICLE3	Diesel Exhausts	[51]
SCT031	SOIL1	Soil Dust	[48]
SCT034	MARIN2	Seawater in South Taiwan	[52]
SCT035	VB001	Biomass Burning	[53]
SCT037	SO4	Secondary Sulfate	[54]
SCT038	NO3	Secondary Nitrate	[54]
SCT039	STONE	Stone Processing Industry	[48]
SCT040	CEMENT2	Cement Industry	[48]
SCT041	CERM1	Ceramic Plants	[48]
SCT042	CERM2	Tile Industry	[48]
SCT043	COAL	Coal Burning	[48]
SCT044	COAA	Coal Ash	[48]
SCT045	SOIL2	Fugitive Dust	[48]
SCT046	VB002	Biomass Burning	[48]
SCT047	CONST	Construction Dust	[48]
SCT048	VEHICLE3	Road Dust	[48]
SCT049	SHIP1	Boat (fishing boat)	[55]
SCT050	DUST	Mineral dust	[56]

Table S3 Dry deposition fluxes of Fe and P in the SCS in this study (μmol m^{-2} d^{-1}).

Types	Samples	Fe	P
Non-dust	0617	1.02	0.60
	0618	0.63	0.43
	0619	0.68	0.79
	0620	2.86	0.90
Dust	0621	9.93	1.52
	0622D	24.95	3.02
	0622N	23.17	3.47
	0623D	15.79	2.34
	0623N	21.81	3.57
	0624D	18.17	2.54
	0624N	18.97	2.53
	0625D	20.46	2.82
	0625N	18.32	2.40
	0626	15.61	1.99
	0627	12.67	1.70
	0628	9.36	1.27
	0629	6.34	0.83

D represented day and N represented night.

References

[1] Luo L, Yao XH, Gao HW, et al. Nitrogen speciation in various types of aerosols in spring over the northwestern Pacific Ocean. *Atmos Chem Phys* 2016; 16(1): 325–41.

[2] Hsu SC, Gong GC, Shiah FK, et al. Sources, solubility, and acid processing of aerosol iron and phosphorous over the South China Sea: East Asian dust and pollution outflows vs. Southeast Asian biomass burning. *Atmos Chem Phys Discuss* 2014; 14(15), 21433–72.

[3] Hsu SC, Liu SC, Huang YT, et al. A criterion for identifying Asian dust events based on Al concentration data collected from northern Taiwan between 2002 and early 2007. *J Geophys Res Atmos* 2008; 113(D18).

[4] Hsu SC, Wong GT, Gong GC, et al. Sources, solubility, and dry deposition of aerosol trace elements over the East China Sea. *Mar Chem* 2010; 120(1-4): 116–27.

[5] Du S, Xiang R, Liu J, et al. The present-day atmospheric dust deposition process in the South China Sea. *Atmos Environ* 2020; 223:117261.

[6] Du S, Ariful Islam GM, Xiang R, et al. The dust deposition process and biogeochemical impacts in the Northern South China Sea. *Asia-Pac J Atmos Sci* 2021; 57: 77–87

[7] Yuan CS, Chuang HL, Tseng YL, et al. Long-range transport and source apportionment of marine fine particles in the Taiwan Strait and South China Sea Intersection: Spatiotemporal variations and chemical fingerprints. *Atmos Environ* 2024; 339, 120867.

[8] Yuan CS, Hung CM, Hung KN, et al. 2023. Route-based chemical significance and source origin of marine PM2.5 at three remote islands in East Asia: spatiotemporal variation and long-range transport. *Atmos Pollut Res* 2023; 14, 101762.

[9] Wu CH, Yuan CS, Yen PH, et al. Diurnal and seasonal variation, chemical characteristics, and source identification of marine fine particles at two remote islands in South China Sea: a superimposition effect of local emissions and long-range transport. *Atmos Environ* 2022; 270, 118889.

[10] Yen PH, Yuan CS, Lee CW, et al. Chemical characteristics and spatiotemporal variation of marine fine particles for clustered channels of air masses transporting toward remote background sites in East Asia. *Environ Pollut* 2023; 331, 121870.

[11] Song J, Zhao Y, Zhang Y, et al. Influence of biomass burning on atmospheric aerosols over the western South China Sea: Insights from ions, carbonaceous fractions and stable carbon isotope ratios. *Environmental Pollution 2018;* 242, 1800-09.

[12] Turpin BJ & Lim HJ. Species contributions to PM2.5 mass concentrations: revisiting common assumptions for estimating organic mass. *Aerosol Sci Tech* 2001; 35, 602-610.

[13] Viidanoja J, Sillanpaa M, Laakia J, et al. Organic and black carbon in PM2.5 and PM10: 1 year of data from an urban site in Helsinki, Finland. *Atmos Environ* 2002; 36, 3183–93.

[14] Andreae MO, Schmid O, Yang H, et al. Optical properties and chemical composition of the atmospheric aerosol in urban Guangzhou, China. *Atmos Environ* 2008; 42, 6335–50.

[15] Xing L, Fu TM, Cao JJ, et al. Seasonal and spatial variability of the OM/OC mass ratios and high regional correlation between oxalic acid and zinc in Chinese urban organic aerosols. *Atmos Chem Phys* 2013; 13, 4307–18.

[16] Crippa M, Canonaco F, Slowik JG, et al. Primary and secondary organic aerosol origin by combined gas-particle phase source apportionment. *Atmos Chem Phys* 2013, 13, 8411–26.

[17] Tiwari S, Pervez S, Cinzia P, et al. Chemical characterization of atmospheric particulate matter in Delhi, India, part II: Source apportionment studies using PMF 3.0. Sustainable Environment Research 2013; 23, 295–306.

[18] Zhang Q, Jimenez JL, Canagaratna MR, et al. Understanding atmospheric organic aerosols via factor analysis of aerosol mass spectrometry: a review. *Anal Bioanal Chem* 2011; 401, 3045–67.

[19] Liang B, Cai M, Sun Q, et al. Source apportionment of marine atmospheric aerosols in northern South China Sea during summertime 2018. *Environ Pollut* 2021; 289: 117948.

[20] Xiao HW, Xiao HY, Luo L, et al. Atmospheric aerosol compositions over the South China Sea: temporal variability and source apportionment. *Atmos Chem Phys* 2017; 17(4), 3199-3214.

[21] Hilario MRA, Cruz MT, Cambaliza MOL, et al. Investigating size-segregated sources of elemental composition of particulate matter in the South China Sea during the 2011 Vasco cruise. *Atmos Chem Phys* 2020; *20*(3), 1255-76.

[22] Yen PH, Yuan CS, Wu CH, et al. Transport route-based cluster analysis of chemical fingerprints and source origins of marine fine particles (PM2. 5) in South China Sea. *Sci Total Environ* 2022; 806, 150591.

[23] Sun Q, Liang B, Cai M, et al. Cruise observation of the marine atmosphere and ship emissions in South China Sea: aerosol composition, sources, and the aging process. *Environ Pollut* 2023; *316*, 120539.

[24] Duce RA, Liss PS, Merrill JT, et al. The atmospheric input of trace species to the world ocean. *Global Biogeochem Cy* 1991; 5: 193–259.

[25] Hoppel WA. Surface source function for sea-salt aerosol and aerosol dry deposition to the ocean surface. *J Geophys Res* 2002; 107: 4832–.

[26] Hsu SC, Liu SC, Arimoto R, et al. Dust deposition to the East China Sea and its biogeochemical implications. *J Geophys Res Atmos* 2009; 114(D15).

[27] Du S, Xiang R, Liu J, et al. The present-day atmospheric dust deposition process in the South China Sea. *Atmos Environ* 2020; 223: 117261.

[28] Baker AR, Kelly SD, Biswas KF, et al. Atmospheric deposition of nutrients to the Atlantic Ocean. *Geophys Res Lett* 2003; 30: 2296.

[29] de Leeuw G, Spokes L, Jickells T, et al. Atmospheric nitrogen inputs into the North Sea: effect on productivity. *Cont Shelf Res* 2003; 23: 1743–55.

[30] Nakamura T, Matsumoto K, Uematsu M. Chemical characteristics of aerosols transported from Asia to the East China Sea: an evaluation of anthropogenic combined nitrogen deposition in autumn. *Atmos Environ* 2005; 39: 1749–58.

[31] Baker AR, Weston K, Kelly SD, et al. Dry and wet deposition of nutrients from the tropical Atlantic atmosphere: Links to primary productivity and nitrogen fixation. *Deep-Sea Res Part I* 2007; 54: 1704–20.

[32] Jung J, Han B, Rodriguez B, et al. Atmospheric Dry Deposition of Water-Soluble Nitrogen to the Subarctic Western North Pacific Ocean during Summer. *Atmosphere* 2019; 10: 15.

[33] Taylor SR, & McLennan SM. The geochemical evolution of the continental crust. *Rev Geophys* 1995; 33(2): 241-65.

[34] Das S, Prospero JM, Chellam S. Quantifying international and interstate contributions to primary ambient $PM_{2.5}$ and PM_{10} in a complex metropolitan atmosphere. *Atmos Environ* 2023; 292: 119415.

[35] Wu, H. Y., Hsieh, C. C., & Ho, T. Y. Trace metal dissolution kinetics of East Asian size-fractionated aerosols in seawater: the effect of a model siderophore. *Mar Chem* 2023; 254, 104277.

[36] Lee CY, Chen HY, Tuo S, et al. Seasonal dynamics of new production from Trichodesmium N2 fixation and nitrate uptake in the upstream Kuroshio and South China Sea basin, *Limnol Oceanogr* 2008; 53(5): 1705-21.

[37] Chen YLL, Chen HY, Lin YH, et al. The relative contributions of unicellular and filamentous diazotrophs to N2 fixation in the South China Sea and the upstream Kuroshio. *Deep-Sea Res Part I* 2014; 85: 56-71.

[38] Voss M, Bombar D, Loick N, et al. Riverine influence on nitrogen fixation in the upwelling region off Vietnam, South China Sea. *Geophys Res Lett* 2006; 33: L07604.

[39] Yang JY, Hsu SC, Dai MH, et al. Isotopic composition of water-soluble nitrate in bulk atmospheric deposition at Dongsha Island: sources and implications of external N supply to the northern South China Sea. *Biogeosciences* 2014; 11(7): 1833-46.

[40] Lu Y, Wen Z, Shi D, et al. Biogeography of N2 fixation influenced by the western boundary current intrusion in the South China Sea. *J Geophys Res Oceans* 2019; 124.

[41] Wu, C., Fu, F. X., Sun, J., et al. Nitrogen fixation by Trichodesmium and unicellular diazotrophs in the northern South China Sea and the Kuroshio in summer. *Sci Rep* 2018; *8*(1), 2415.

[42] Dong, J., Zhang, Y., Wang, Y., et al. Spatial and seasonal variations of Cyanobacteria and their nitrogen fixation rates in Sanya Bay, South China Sea. *Scientia Marina 2008; 72*(2), 239-51.

[43] Li, L., Wu, C., Sun, J., et al. Nitrogen fixation driven by mesoscale eddies and the Kuroshio Current in the northern South China Sea and the East China Sea. *Acta Oceanol Sin* 2020; *39*, 30-41.

[44] Kao SJ, Terence Yang JY, Liu KK, et al. Isotope constraints on particulate nitrogen source and dynamics in the upper water column of the oligotrophic South China Sea. *Glob Biogeochem Cycles* 2012; 26(2).

[45] Liu, J., Zhou, L., Li, J., et al. Effect of mesoscale eddies on diazotroph community structure and nitrogen fixation rates in the South China Sea. *Reg Stud Mar Sci* 2020; *35*, 101106.

[46] Chow JC, Watson JG, Kuhns H, et al. Source profiles for industrial, mobile, and area sources in the Big Bend Regional Aerosol Visibility and Observational study. *Chemosphere* 2004; 54: 185–208.

[47] Yang H, Arafath SM, Lee K, et al. Chemical characteristics of filterable and condensable PM2.5 emissions from industrial boilers with five different fuels. *Fuel* 2018; 232: 415–22.

[48] Li T, Wu C, Chen W, et al. Diurnal Variation and Chemical Characteristics of Atmospheric Aerosol Particles and Their Source Fingerprints at Xiamen Bay. *Aerosol Air Qual Res* 2013; 13: 596–607.

[49] Hsu C, Chiang H, Lin S, et al. Elemental characterization and source apportionment of PM 10 and PM 2.5 in the western coastal area of central Taiwan. *Sci Total Environ* 2016; 541: 1139–50.

[50] Cheng Y, Li Y. Influences of Traffic Emissions and Meteorological Conditions on Ambient PM10 and PM2.5 Levels at a Highway Toll Station. *Aerosol Air Qual Res* 2010; 10: 456–62.

[51] Watson SA, Huang W, Wong VW. Correlations among ash-related oil species in the power cylinder, crankcase and the exhaust stream of a heavy-duty diesel engine (No. 2007-01-1965). *SAE Tech Pap* 2007.

[52] Li T, Yuan C, Huang H, et al. Clustered long-range transport routes and potential sources of PM 2.5 and their chemical characteristics around the Taiwan Strait. *Atmos Environ* 2017; 148: 152–66.

[53] Sheesley RJ, Schauer JJ, Zheng M, et al. Sensitivity of molecular marker-based CMB models to biomass burning source profiles. *Atmos Environ* 2007; 41: 9050–63.

[54] Wang X, Bi X, Sheng G, et al. Chemical Composition and Sources of PM10 and PM2.5 Aerosols in Guangzhou, China. *Environ Monit Assess* 2006; 119: 425–39.

[55] Yuan C, Wong K, Tsengn Y, et al. Chemical significance and source apportionment of fine particles (PM2.5) in an industrial port area in East Asia. *Atmos Pollut Res* 2022; 13: 101349.

[56] Bozlaker A, Prospero JM, Fraser MP, et al. Quantifying the Contribution of Long-Range Saharan Dust Transport on Particulate Matter Concentrations in Houston, Texas, Using Detailed Elemental Analysis. *Environ Sci Technol* 2013; 47(18): 10179–87.
